# Sensitizing Triple Negative Breast Cancer to Tamoxifen Chemotherapy via a Redox-Responsive Vorinostat-containing Polymeric Prodrug Nanocarrier

**DOI:** 10.7150/thno.38973

**Published:** 2020-01-20

**Authors:** Weina Ma, Jingjing Sun, Jieni Xu, Zhangyi Luo, Dingwei Diao, Ziqian Zhang, Patrick J. Oberly, Margaret Beth Minnigh, Wen Xie, Samuel M Poloyac, Yi Huang, Song Li

**Affiliations:** 1 Center for Pharmacogenetics,; 2 Department of Pharmaceutical Sciences, School of Pharmacy, University of Pittsburgh, Pittsburgh, PA, 15261, USA;; 3 Department of Pharmacology and Chemical Biology,; 4 UPMC Hillman Cancer Center, School of Medicine, University of Pittsburgh, Pittsburgh, PA, 15213, USA; 5 Current address: School of Pharmacy, Health Science Center, Xi'an Jiaotong University, Xi'an 710061, P.R. China.

**Keywords:** vorinostat, prodrug micelles, redox responsive, tamoxifen, co-delivery

## Abstract

There is an urgent and unmet need to develop effective therapies for triple negative breast cancers (TNBCs) which are much more aggressive and have poor prognosis due to lack of receptor targets for Her2-targeted and endocrine therapy. In this study we systematically evaluated the effect of Vorinostat (SAHA, a pan-HDAC inhibitor) in reactivating the expression of functional estrogen receptor α (ERα) and synergizing with tamoxifen (TAM, a selective estrogen-receptor modulator) in antitumor activity. In addition, a SAHA prodrug-based dual functional nanocarrier was developed for codelivery of SAHA and TAM for effective combination therapy.

**Methods**: A SAHA-containing polymeric nanocarrier, POEG-*co*-PVDSAHA was developed via reversible addition-fragmentation transfer (RAFT) polymerization with SAHA incorporated into the polymer through a redox-responsive disulfide linkage. The effect of both free SAHA and POEG-*co*-PVDSAHA on reactivating the expression of functional ERα was investigated in several human and murine TNBC cell lines via examining the mRNA and protein expression of ERα target genes. The cytotoxicity of free SAHA and TAM combination and TAM-loaded POEG-*co*-PVDSAHA micelles was examined via MTT assay. The *in vivo* antitumor activity of TAM-loaded POEG-*co*-PVDSAHA was investigated in a murine breast cancer model (4T1.2).

**Results**: Both free SAHA and POEG-*co*-PVDSAHA were effective in inducing the reexpression of functional estrogen receptor α (ERα), which may have helped to sensitize TNBCs to TAM. More importantly, POEG-*co*-PVDSAHA self-assembled to form small-sized micellar carrier that is effective in formulating and codelivery of TAM. TAM-loaded POEG-*co*-PVDSAHA micelles exhibited enhanced and synergistic cytotoxicity against TNBC cell lines compared with free SAHA, free TAM and TAM loaded into a pharmacologically inert control carrier (POEG-*co*-PVMA). In addition, codelivery of TAM via POEG-*co*-PVDSAHA micelles led to significantly improved antitumor efficacy in 4T1.2 tumor model compared with other groups such as combination of free SAHA and TAM and TAM-loaded POEG-*co*-PVMA micelles.

**Conclusion**: Our prodrug-based co-delivery system may provide an effective and simple strategy to re-sensitize TNBCs to TAM-based hormone therapy.

## Introduction

Chemotherapy remains one of the most widely used therapeutic strategies for cancers^1^. However, conventional monotherapy based on a single chemotherapeutic drug has major limitations, such as undesirable adverse effects, and drug resistance^2^, ^3^. To overcome these limitations, combination therapy with multiple anticancer drugs of different pharmacological mechanisms is becoming a promising strategy for cancer treatment^4^-^7^.

Histone deacetylases (HDACs) are enzymes which can deacetylate lysine residues in histones and can be divided into Zn^2+^-dependent classes (class I, II and IV) and NAD-dependent classes (class III) according to the functional criteria and the homology to yeast proteins^8^. As the functional antagonists of histone acetyltransferases (HATs), HDACs remove the acetyl groups in order to make chromatin structure compressed and suppress gene transcription 9. Some reports show that HDACs can impact the tumor growth and overexpression of HDACs has been observed in several types of cancers, such as breast cancer, prostate cancer and colon cancer^10^. HDAC inhibitors can block the de-acetylation of histone, thus modifying chromatin structure and relieving transcriptional repression^11^, ^12^. HDAC inhibitors are effective in treating a variety of cancers in clinical trials, such as belinostat for peripheral T-cell lymphoma and panobinostat for multiple myeloma^13^. HDACs also play an important role in estrogen receptor (ER) gene silencing in ER-negative breast cancer cells, especially the HDAC1 which is associated with ER expression^11^. Reports have shown that inhibition of HDAC can reactivate the expression of functional ER in ER-negative breast cancer cells^11^ and re-sensitize the ER-negative breast cancer cells to tamoxifen (TAM), indicating that the combination with HDAC inhibitors will render antiestrogens-based endocrine therapy a new viable treatment for triple negative breast cancers (TNBCs)^14^, ^15^.

Vorinostat (suberoylanilide hydroxamic acid, SAHA) is a pan-HDAC (class I and II) inhibitor approved by US Food and Drug Administration (FDA) for the treatment of advanced, refractory cutaneous T-cell lymphoma^16^. SAHA acts as a chelator for the zinc ions in the active site of HDACs and is considered as a promising chemotherapeutic drug against cancers^17^. The preclinical applications of SAHA to various cancers, such as breast cancer, lung cancer and prostate cancer, as mono-chemotherapy or in combination with other therapies, have been widely investigated^18^-^21^. SAHA has been shown to inhibit growth of both ER-negative and ER-positive breast cancer cell lines^11^. Furthermore, the efficacy of TAM could be enhanced by SAHA and this combination had no unexpected untoward effect in patients with hormone-resistant breast cancer^4^. In addition, some studies reported that SAHA could re-sensitize the TAM-resistant cells to hormone therapy^22^ and could reactivate functional ER expression in ER-negative breast cancer cell lines^14^. TNBCs that lack the expression of ER, progesterone receptor (PR), and human epidermal growth factor 2 (HER2) can hardly respond to hormonal therapy (TAM)^23^. All the studies provide the strong rational for combining SAHA with TAM as a new and improved therapy for TNBCs^14^, ^22^, ^24^. The major challenges that limit the success of such combination therapy are the limited oral bioavailability and poor stability of SAHA and the difficulty in achieving effective codelivery of the two agents to the tumor sites.

Nanotechnology represents a promising approach for tumor-selective delivery as well as codelivery of several different anticancer agents and various nanodelivery systems have been reported 25-31. These include nanocarriers for delivery of SAHA or TAM alone^32^-^36^. One promising nanocarrier is based on the PEG-hydrophobic drug conjugates (prodrugs) that have both the function of delivery and the intrinsic antitumor activity^26^, ^37^-^40^. In addition, this approach can provide controlled temporal-spatial release of two drugs through both chemical conjugation and physical encapsulation^41^. In this study, the impact of SAHA on several TNBC cell lines and the potential combination therapy with TAM for TNBC were investigated. More importantly, we have designed and synthesized a redox-responsive SAHA-based prodrug polymer with a disulfide linkage between SAHA and polymer backbone, denoted by POEG-*co*-PVDSAHA. We examined the potency of POEG-*co*-PVDSAHA in reactivating the expression of ER in comparison with free SAHA. The potential of POEG-*co*-PVDSAHA in codelivery of and synergistic action with TAM was also examined both *in vitro* and *in vivo*.

## Materials and methods

### Materials

Vorinostat (SAHA) and tamoxifen (TAM) were purchased from LC Laboratories (Woburn, MA, USA). Phenol red-free dulbecco's modified eagle medium (DMEM) and penicillin-streptomycin solution were purchased from Invitrogen (Carlsbad, CA, USA) and dextran-charcoal-stripped fetal bovine serum (FBS) was from Atlanta Biologicals (Lawrenceville, GA, USA). 3-(4,5-dimethylthiazol-2-yl)-2,5-diphenyl tetrazolium bromide (MTT), Dimethyl sulfoxide (DMSO) and trypsin-EDTA solution were obtained from Sigma-Aldrich (St. Louis, MO, USA). Dulbecco's phosphate buffered saline (DPBS) was purchased from Invitrogen (Carlsbad, CA, USA). RIPA Lysis Buffer and RNAfast200 kit were purchased from Pierce Biotechnology (Waltham, MA, USA). High Capacity cDNA Reverse Transcription Kit, SYBR green and Pierce^TM^ ECL Western Blotting Substrate were purchased from Thermo Fisher Scientific Inc. (Waltham, MA, USA). Immun-Blot^®^ polyvinylidene fluoride (PVDF) membrane was obtained from Bio-Rad Laboratories, Inc. (Hercules, CA, USA). The ERα monoclonal antibody (working dilution: 1:1000) was purchased from Thermo Fisher Scientific Inc. (Waltham, MA, USA). The GAPDH and β-actin monoclonal antibodies (1:1000 dilution) were purchased from Cell Signaling Technology (Danvers, MA, USA). HRP-conjugated goat anti-rabbit IgG was purchased from Pierce Biotechnology (Waltham, MA, USA). Aspartate aminotransferase (AST)/SGOT LIQUI-UV^®^ and alanine transaminase (ALT)/SGOT LIQUI-UV^®^ were purchased from Stanbio Laboratory (Boerne, TX, USA). QuantiChrom^TM^ Creatinine Assay Kit was obtained from BioAssay Systems (Hayward, CA, USA). Lipofectamine^®^ 2000 Reagent was purchased from Invitrogen (Carlsbad, CA, USA). Bright-Glo Luciferase Assay System was from Promega (Madison, WI, USA). All other reagents used in this study were analytical or HPLC grade.

### Synthesis of POEG-*co*-PVDSAHA polymer

POEG-*co*-PVD polymer backbone was synthesized by reversible addition-fragmentation transfer (RAFT) polymerization 42. AIBN (3 mg), 4-Cyano-4-(thiobenzoylthio)pentanoic acid (6 mg), VD monomer (200 mg), OEG950 monomer (400 mg), and 1 mL of dried tetrahydrofuran were added into a Schlenk tube. After three free-pump-thawing cycles, the mixture was stirred at 80 °C under the protection of N_2_. After 18 h, the reaction mixture was precipitated in ether to give the POEG-*co*-PVD polymer. SAHA was conjugated to the POEG-*co*-PVD polymer by adding SAHA (80 mg) into the DMSO solution of POEG-*co*-PVD (60 mg), DCC (150 mg) and DMAP (10 mg). The mixture was stirred at room temperature for 24 h and then dialyzed against DMSO and water for 2 days. After centrifugation, the supernatant was lyophilized to give POEG-*co*-PVDSAHA polymer.

### Synthesis of POEG-*co*-PVMA polymer

POEG-*co*-PVBC polymer backbone was synthesized by RAFT copolymerization of OEG950 and VBC monomers. OEG950 (900 mg), VBC monomer (600 mg), 4-Cyano-4-(phenylcarbonothioylthio) pentanoic acid (12 mg), AIBN (4 mg), and 1 mL of dried tetrahydrofuran were added into a Schlenk tube. After three free-pump-thawing cycles, the mixture was stirred at 80 °C under the protection of N_2_. After 18 h, the reaction mixture was precipitated in ether to give the POEG-*co*-PVBC polymer. Myristic acid was conjugated to the POEG-*co*-PVBC polymer by adding myristic acid (200 mg) into the DMF mixture of POEG-*co*-PVBC (100 mg) and K_2_CO_3_ (200 mg). The mixture was stirred at 50 °C for 15 h and then dialyzed against DMSO and water for 2 days. After centrifugation, the supernatant was lyophilized to give POEG-*co*-PVMA polymer.

### Preparation and characterization of micelles

The drug-loaded micelles were prepared by mixing TAM (5 mg/mL in dichloromethane) with POEG-*co*-PVDSAHA or POEG-*co*-PVMA polymer (50 mg/mL in dichloromethane) at the indicated carrier/drug ratios. The organic solvent was removed by N_2_ flow to form a thin film of drug/carrier mixture. The film was then further dried under vacuum for 1 h and saline was added to form the drug-loaded micelles. The particle sizes of blank and drug-loaded micelles were measured by a Zetasizer from Malvern Panalytical Inc. (Westborough, MA, USA). The morphologies of both blank and drug-loaded micelles were examined by transmission electron microscopy (TEM) using negative staining method.

### Critical micelle concentration (CMC) of micelles

The CMC values of POEG-*co*-PVDSAHA and POEG-*co*-PVMA micelles were evaluated by fluorescence assay with nile red as a fluorescence probe. Briefly, 30 μL of nile red solution (0.05 mg/mL) in dichloromethane (DCM) was added to the test tubes and then the solvent was removed by evaporation at room temperature. Then, 2 mL of POEG-*co*-PVDSAHA and POEG-*co*-PVMA micelles ranging from 1×10^-4^ to 5×10^-1^ mg/mL was added to each tube with nile red respectively. The solution was kept overnight to ensure that nile red reached the solubilization equilibrium. The fluorescence was measured at 570~720 nm with an excitation wavelength of 550 nm.

### Disassembly of blank micelles triggered by glutathione (GSH)

The disassembly of redox-sensitive POEG-*co*-PVDSAHA micelles in response to different concentrations of GSH was examined by a Zetasizer to monitor the size changes of the micelles. Briefly, 2 mL of POEG-*co*-PVDSAHA micelle solution containing GSH (10 μM and 10 mM) was placed in an incubation shaker at 37 °C at a speed of 100 rpm for 4 h. For comparison, POEG-*co*-PVDSAHA micelles incubated without GSH and redox-non-sensitive POEG-*co*-PVMA micelles with and without 10 mM GSH were used as controls.

### *In vitro* release

The kinetics of TAM release from TAM-loaded POEG-*co*-PVDSAHA and POEG-*co*-PVMA micelles at different concentrations of GSH was examined by a dialysis method. Briefly, 1 mL of TAM/POEG-*co*-PVDSAHA micelles (1 mg TAM/mL) was placed into a dialysis bag (MWCO, 3.5 KDa), and then incubated in 50 mL PBS containing 0.5% (w/v) Tween 80 and different concentrations of GSH (0, 10 μM and 10 mM) with shaking gently at 37 °C at 100 rpm. For comparison, free TAM and TAM/POEG-*co*-PVMA micelles were incubated with and without 10 mM GSH. At different time intervals, the TAM and SAHA concentrations were examined by HPLC at 280 nm and 245 nm wavelength, respectively.

### Cell culture

4T1.2 cells and MDA-MB-231 cells were cultured in phenol red-free DMEM medium containing 10% fetal bovine serum, 100 U/mL penicillin, and 100 U/mL streptomycin. HS578T cells were cultured in phenol red-free DMEM medium containing 10% fetal bovine serum, 100 U/mL penicillin, and 100 U/mL streptomycin, and 0.01 mg/mL bovine insulin. Cells were grown at 37 °C in a humidified atmosphere with 5% CO_2_. All the cell lines were obtained from ATCC (Manassas, VA, USA).

### Animals

Female BALB/c mice, 4-6 weeks in age, were obtained from Charles River (Davis, CA, USA). All animals were housed under pathogen-free conditions according to AAALAC guidelines. All animal-related experiments were performed in full compliance with institutional guidelines and approved by the Animal Use and Care Administrative Advisory Committee at the University of Pittsburgh.

### RT-PCR and quantitative real-time PCR

Total RNA was extracted using the RNAfast200 kit according to the manufacturer's protocol. The RT-PCR was performed using High Capacity cDNA Reverse Transcription Kit. Real-time PCR was performed using SYBR green as a fluorescence probe. The primer sequences were as following: GAPDH forward primer (Mouse): 5'-AGGTTGTCTCCTGCGACTTCA-3'; GAPDH reverse primer (Mouse): 5'-TGGTCCAGGGTTTCTTACTCC-3'; GAPDH forward primer (Human): 5'-GCACCGTCAAGGCTGAGAAC-3'; GAPDH reverse primer (Human): 5'-TGGTGAAGACGCCAGTGGA-3'; ER-α forward primer (Mouse): 5'-GCCCTCCCGCCTTCTACA-3'; ER-α reverse primer (Mouse): 5'-CCCTCCTCGGCGGTCTTT-3'; ER-α forward primer (Human): 5'-TGTGCCTGGCTAGAGATCCTGA-3'; ER-α reverse primer (Human): 5'-AGCCAGCAGCATGTCGAAGA-3'; PGR forward primer (Mouse): 5'-TCCCCCCACTGATCAACTTG-3'; PGR reverse primer (Mouse): 5'-TCCGAAAACCTGGCAGTGA-3'; PGR forward primer (Human): 5'-TGGAAGAAATGACTGCATCG-3'; PGR reverse primer (Human): 5'-TAGGGCTTGGCTTTCATTTG-3'.

### Western blot analysis

Cells and tissue samples were lysed in RIPA lysis buffer at 4 °C for 10 min. The lysates were centrifuged (12,000 g) at 4 °C for 10 min. An equivalent amount of protein was resolved by 10% SDS-PAGE and transferred to PVDF membranes. The membranes were then blocked in 5% non-fat powdered milk dissolved in Tris-buffer saline containing 0.05% Tween-20 (TBST) for 1 h. Afterwards, the blot was incubated with the blocking solution containing primary antibody overnight at 4 °C. After washing with TBST for 5 min three times, the blot was incubated with a secondary antibody at room temperature for 1 h. The blot was then washed with TBST three times for 5 min each before being exposed to the SuperSignal West Dura Extended Duration substrate.

### Luciferase assay

Exponential-phase cells were plated into 48-well plates in complete medium. After 24 h, cells were transfected with estrogen response element (ERE) reporter and β-gal plasmid using the Lipofectamine^®^ 2000 transfection reagent for 6 h. Then, cells were treated with different concentrations of indicated drugs for 24 h. Afterwards, cells were stimulated with estrogen for 6 h before the luciferase assay. Finally, luciferase activities were determined by Bright-Glo Luciferase Assay System. Relative reporter activity was calculated by comparing with the vehicle-treated group. All transfections were performed in triplicates.

### *In vitro* cytotoxicity assay

Exponential-phase cells were plated into 96-well plates in complete medium. After 24 h, cells were treated with indicated drugs at different concentrations and incubated for 48 h. One hundred μL MTT solution (0.5 mg/mL) dissolved in saline were then added to each well. Plates were incubated for 4 h at 37 °C. After removing the MTT solution, 100 μL DMSO was added to each well. The absorbance was recorded at a wavelength of 490 nm in a micro-plate reader.

### Near-infrared fluorescence optical imaging

Two hundred μL of Dir/POEG-*co*-PVMA micelles or Dir/POEG-*co*-PVDSAHA micelles with a DiR concentration of 0.4 mg/mL were injected into BALB/c mice bearing s.c. 4T1.2 xenografts. At specific times, the mice were anesthetized by isoflurane inhalation and imaged by Multispectral FX PRO system (Carestream Molecular Imaging) with exposure for 60 s at an excitation of 730 nm and an emission of 835 nm. After 24 h, the mice were euthanized by CO_2_ overdose. The tumor and other organs were collected for *ex vivo* imaging.

### Biodistribution of TAM and SAHA

TAM-loaded POEG-co-PVDSAHA micelles were injected into 4T1.2 tumor-bearing mice at a TAM dose of 10 mg/kg. After 24 h, the mice were sacrificed to collect the major organs. The tissues were weighed and homogenized in saline water (triple in weight) with 100 mM DTT. Then same volume of acetonitrile was added to the homogenized sample and the solution was mixed via sonication. The samples were centrifuged at 3500 rpm for 10 min, and 200 μL supernatants were collected and dried under airflow. The residues were then re-dissolved in 200 μL of solvent (acetonitrile:H_2_O=1:1, v/v) and centrifuged at 12500 rpm for 10 min. Quantitation of TAM and SAHA in the clear supernatants was achieved by eluting the compounds from a Waters Acquity UPLC BEH C18, 1.7 um, 2.1x100 mm reversed phase column, with an acetonitrile:water (0.1%formic acid) gradient at 0.3 ml/min. The gradient started from 80% acetonitrile to 5% acetonitrile over 2.5 min where it remained for 2.5 min, and then increased to 80% over 1 min. Detection and quantitation were achieved in the positive mode with a Thermo Fisher TSQ Quantum Ultra mass spectrometer interfaced via an electrospray ionization (ESI) probe. MS Detection conditions were optimized as follows: spray voltage (3000 V), capillary temperature (300 °C), and collision gas pressure (1.5 mTorr). Transitions used for analysis are 327.2 → 72.1 for TAM and 265.2 → 232.2 for SAHA. The lower limit of quantitation is 0.4 ng/ml.

### *In vivo* therapeutic efficacy

Female BALB/c mice (4-6 weeks) were s.c. inoculated with 4T1.2 cells at a density of 2× 10^5^ cells per mouse. When the tumor volume reached about 50 mm^3^, mice were randomly grouped (n=5) and treated with saline, free SAHA, free TAM, combination of free SAHA and TAM, POEG-*co*-PVMA micelles, TAM/POEG-*co*-PVMA micelles, POEG-*co*-PVDSAHA micelles and TAM/POEG-*co*-PVDSAHA micelles (10 mg TAM per kg), respectively once every 3 days for 7 times by tail vein injection. Tumor volumes were monitored and calculated according to the formula: (L*W^2^)/2 (L and W are the long and short tumor diameters). Relative tumor volume was used to compare different groups. Body weights were also followed throughout the entire treatment period.

After completing the *in vivo* experiment, tumor tissues were collected, fixed in 10% formaldehyde, and then embedded in paraffin. The paraffin-embedded tumor tissues were sectioned into slices at 4 μm using an HM 325 Rotary Microtome.

### *In vivo* toxicity assay

Whole blood was taken out of the eye socket of the mice after completing the *in vivo* experiment and put into the 1.5 mL tubes pretreated with heparin. Then the blood samples were centrifuged at 12,000 g at 4 °C for 10 min, and serum was collected for examinations of AST, ALT and creatinine according to the manufacturer's protocol.

### Ki67 staining

For immunochemistry assay, the tumor tissue sections were deparaffinized in xylene and hydrated in descending grades of ethyl alcohol. Then, the sections were pretreated with a boiling 0.1 M sodium citrate buffer in 10% ethyl alcohol and incubated with 0.3% (v/v) hydrogen peroxide to inactivate endogenous peroxidase activity. Then, the sections were washed twice in distilled water and incubated with diluted normal blocking serum for 1 h. After that, the sections were incubated with primary antibody diluted in blocking buffer at 4 ˚C overnight and washed with TBST for three times before incubating with secondary antibody. Then the sections were washed with TBST and treated with Vectastain Elite ABC reagent. The sections were incubated with DAB substrate at room temperature for 15 s. Finally, counterstaining was conducted with hematoxylin for imaging under a BZ-X710 Fluorescence Microscope (Keyence, Itasca, IL, USA).

### Statistical analysis

All values were expressed as means ± standard error of means (SEM). Statistics was determined with ANOVA. Results were considered statistically significant if the P value was < 0.05.

## Results

### SAHA treatment led to re-expression of ER and sensitization to estrogen and TAM

**Figure 1:**
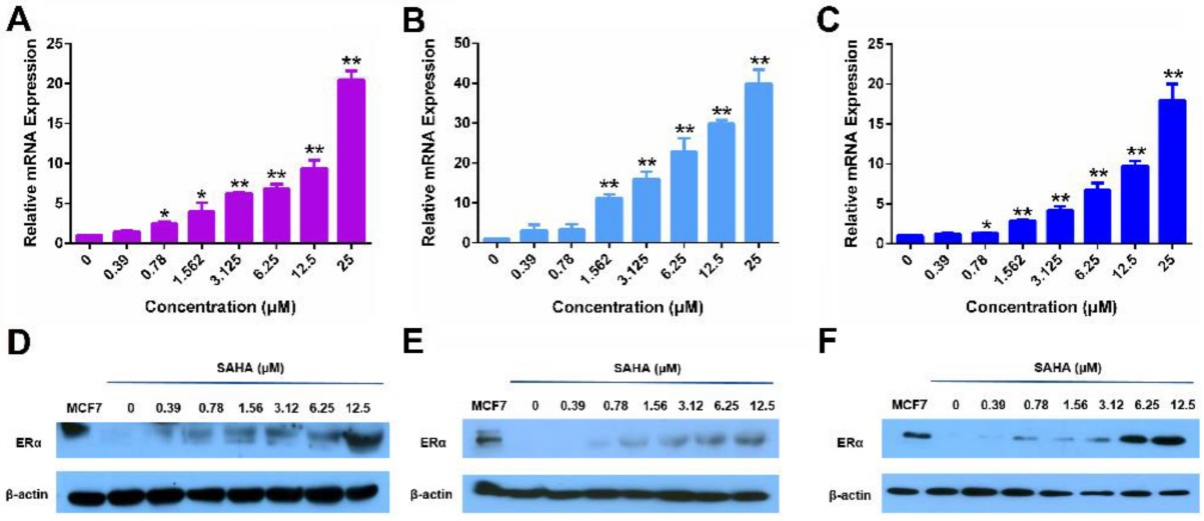
** The re-expression of ERα induced by free SAHA in TNBC cells.** The expression of ERα mRNA induced by free SAHA in MDA-MB-231 cells (A), HS578T cells (B) and 4T1.2 cells (C). Effect of free SAHA micelles on ERα protein level in MDA-MB-231 cells (D), HS578T cells (E) and 4T1.2 cells (F). The concentrations of TAM are 0, 0.39, 0.78, 1.562, 3.125, 6.25, 12.5 and 25 µM. Data represents the means ± SEM (n=3). * p< 0.05, ** *p*< 0.01 versus the control group.

Figure 1 shows that treatment of MDA-MB-231 cells, a human TNBC cell line, with SAHA led to increased expression of ERα at both mRNA (Panel A) and protein (Panel D) levels in a dose-dependent manner. Similar results were obtained in another human TNBC cell line (HS578T cells, Panels B/E) and a murine TNBC cell line (4T1.2 cells, Panels C/F). These results were consistent with a previous report 43.

**Figure 2:**
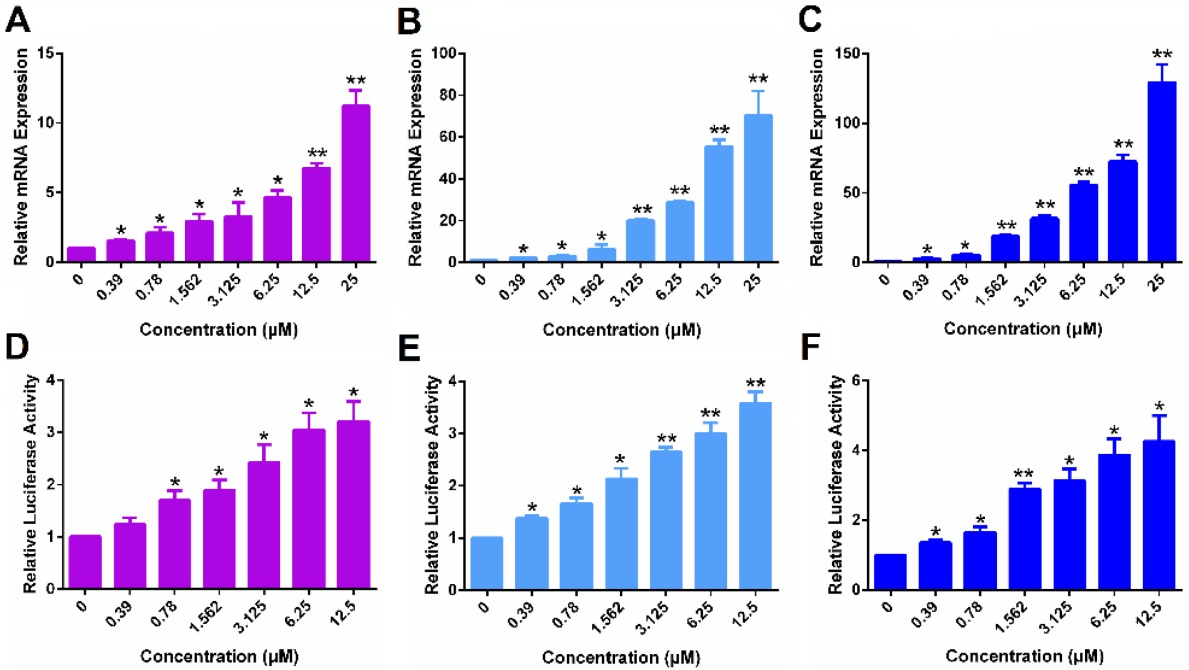
** The function of ERα re-activated by free SAHA in TNBC cells.** The expression of PGR mRNA induced by free SAHA in MDA-MB-231 cells (A), HS578T cells (B) and 4T1.2 cells (C). Effect of free SAHA on induction of an *ERE*(3)-luciferase reporter in MDA-MB-231 cells (D), HS578T cells (E) and 4T1.2 cells (F). The concentrations of SAHA are 0, 0.39, 0.78, 1.562, 3.125, 6.25 and 12.5 µM. Data represents the means ± SEM (n=3). * p< 0.05, ** *p*< 0.01 versus the control group.

Following demonstration of ERα reexpression by SAHA, we then examined whether the re-expressed ERα is functional. Progesterone receptor gene (PGR) is a known target gene of ER. As shown in Figure 2A, treatment with SAHA led to increased mRNA expression of PGR in MDA-MB-231 cells in a dose-dependent manner. Figure 2D shows the induction of luciferase reporter expression in MDA-MB-231 cells that are transfected with an *ERE*(3)-luciferase reporter. Cells were then treated with various concentrations of SAHA for 24 h followed by treatment with estrogen (E2) for another 6 h. It is apparent that SAHA treatment similarly induced the expression of luciferase reporter in a dose-dependent manner (Figure 2D). Similar induction of PGR and luciferase reporter by SAHA was observed in HS578T cells (Figure 2B & E) and 4T1.2 cells (Figure 2C & F). The above data clearly suggest that the re-expressed ERα following SAHA treatment is functional.

**Figure 3:**
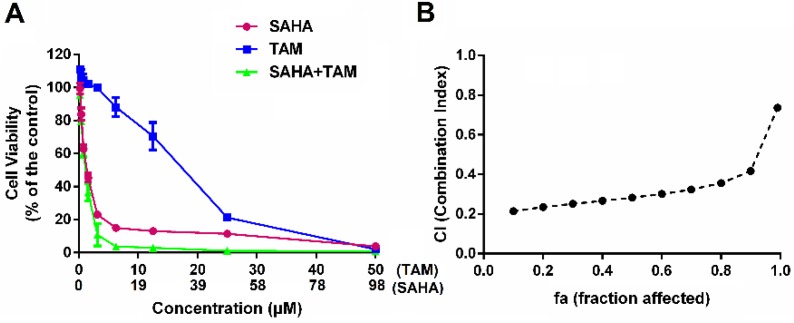
** Synergistic antitumor effect between SAHA and TAM on 4T1.2 cell proliferation.** (A) Dose-response study of a fixed ratio combination of SAHA (0, 0.742, 1.484, 2.969, 5.938, 11.875, 23.75, 47.5 and 98 μM) and TAM (0, 0.39, 0.78, 1.562, 3.125, 6.25, 12.5, 25 and 50 μM) against 4T1.2 cells. (B) fa-CI plot in which fa and CI indicate fraction affected and combination index, respectively. CI<1, CI=1, and CI>1 denote synergistic, additive, and antagonistic interaction, respectively. Each data represents the means ± SEM of triplicate experiments.

Figure 3A shows the inhibitory effect of SAHA and TAM, alone or in combination, on 4T1.2 cells as determined by the MTT assay. SAHA inhibited the proliferation of 4T1.2 cells in a dose-dependent manner. At concentrations below 12.50 µM, TAM alone showed minimal impact on cell proliferation, which is consistent with the notion that TAM was not effective for breast cancer cells that lack ER expression. However, combination with SAHA of a wide concentration range, particularly from 0.39 to 3.12 µM, led to a significant improvement in inhibition of tumor cell proliferation. TAM alone showed significant impact on the proliferation of 4T1.2 cells at concentrations above 12.50 µM, likely due to off-target effect at high concentrations. Nonetheless, combination with SAHA led to further improvement in antitumor activity. Combination index (CI) was then calculated to assess a potential synergy between SAHA and TAM. All of CI values listed in Figure 3B were <1, indicating the synergy between SAHA and TAM. Similar results were obtained in MDA-MB-231(Figure S1) and HS578 cell lines (Figure S2).

### Characterization of POEG-*co*-PVDSAHA and POEG-*co*-PVMA polymers

**Figure 4:**
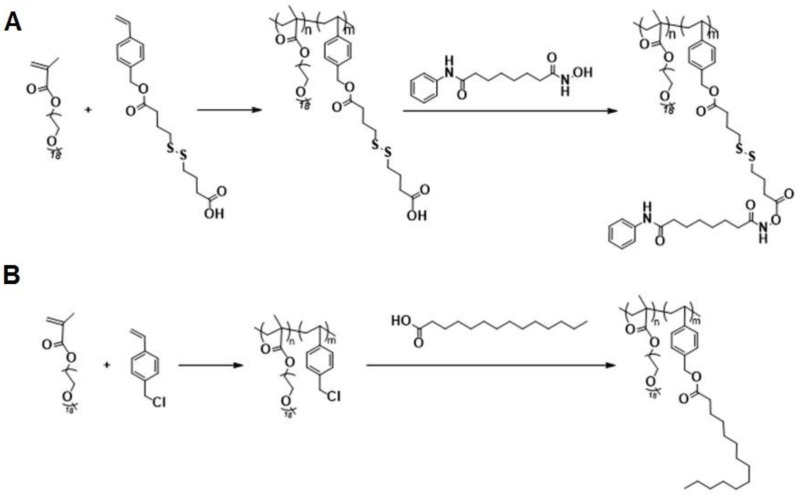
Synthesis routes for POEG-*co*-PVDSAHA (A) and POEG-*co*-PVMA (B) polymers via RAFT polymerization and post-conjugation.

The above data suggest that combination of SAHA and TAM may represent a new therapy for TNBCs. As a strategy to improve the stability of SAHA and facilitate selective codelivery of SAHA and TAM to tumor tissues, an amphiphilic polymer (POEG-*co*-PVDSAHA) with built-in units of SAHA was developed (Figure 4A). The POEG-*co*-PVDSAHA was synthesized via RAFT polymerization and subsequent post-conjugation of SAHA with a disulfide linkage. Therefore, POEG-*co*-PVDSAHA is a SAHA prodrug that is designed to facilitate release of SAHA upon exposure to the highly reductive tumor microenvironment. We also synthesized a pharmacologically “inert” carrier (POEG-*co*-PVMA) as a control carrier, to which myristic acid was conjugated instead (Figure 4B). The structures of POEG-*co*-PVDSAHA and POEG-*co*-PVMA polymer were characterized by ^1^H NMR (Figure S3). According to the intensities of *I*_a_, *I*_b_ and *I*_c_, there are 10 units of OEG950 and 18 units of VD monomer in the POEG-*co*-PVD polymer, and the number of SAHA units conjugated to the POEG-*co*-PVD polymer was determined to be 4. Accordingly, a SAHA loading in the POEG-*co*-PVDSAHA polymeric carrier was calculated to be 13.9% (w/w). Figure S4 shows the ^1^H NMR spectrum of POEG-*co*-PVMA polymer. By comparing the intensities of *I*_b_ and *I*_c_ with *I*_a_, the number of VBC units and the conjugated myristic acid units were determined to be 22 and 18, respectively. The weight fraction of hydrophobic moieties in POEG-*co*-PVMA polymer is similar to that in POEG-*co*-PVDSAHA polymer, making it a suitable control polymer.

**Figure 5:**
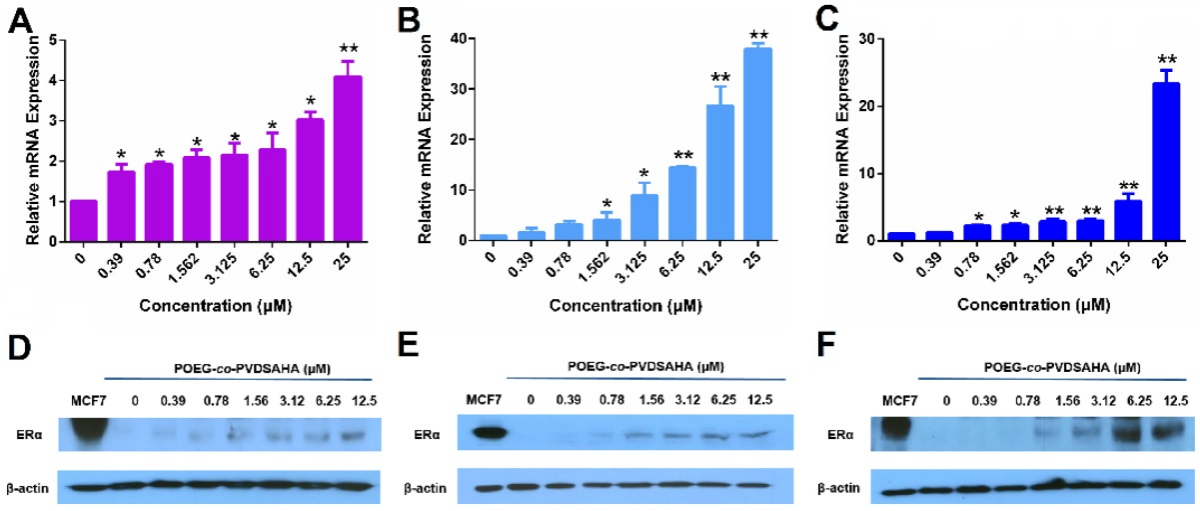
** The re-expression of ERα induced by POEG-*co*-PVDSAHA micelles in TNBC cells.** The expression of ERα mRNA induced by POEG-*co*-PVDSAHA micelles in MDA-MB-231 cells (A), HS578T cells (B) and 4T1.2 cells (C). Effect of POEG-*co*-PVDSAHA micelles on ERα protein level in MDA-MB-231 cells (D), HS578T cells (E) and 4T1.2 cells (F). The concentrations of SAHA in the micelles are 0, 0.39, 0.78, 1.562, 3.125, 6.25, 12.5 and 25 µM. Data represents the means ± SEM (n=3). * *p*< 0.05, ** *p*< 0.01 versus the control group.

Figure 5A & D show that POEG-*co*-PVDSAHA induced the expression of ERα at both mRNA (A) and protein (D) levels in a dose-dependent manner in MDA-MB-231 cells. The control POEG-*co*-PVMA polymer showed no effect on the expression on ERα at both mRNA and protein levels (data not shown). Similar results were shown in HS578 (Figure 5B & E) and 4T1.2 (Figure 5C & F) cell lines.

**Figure 6:**
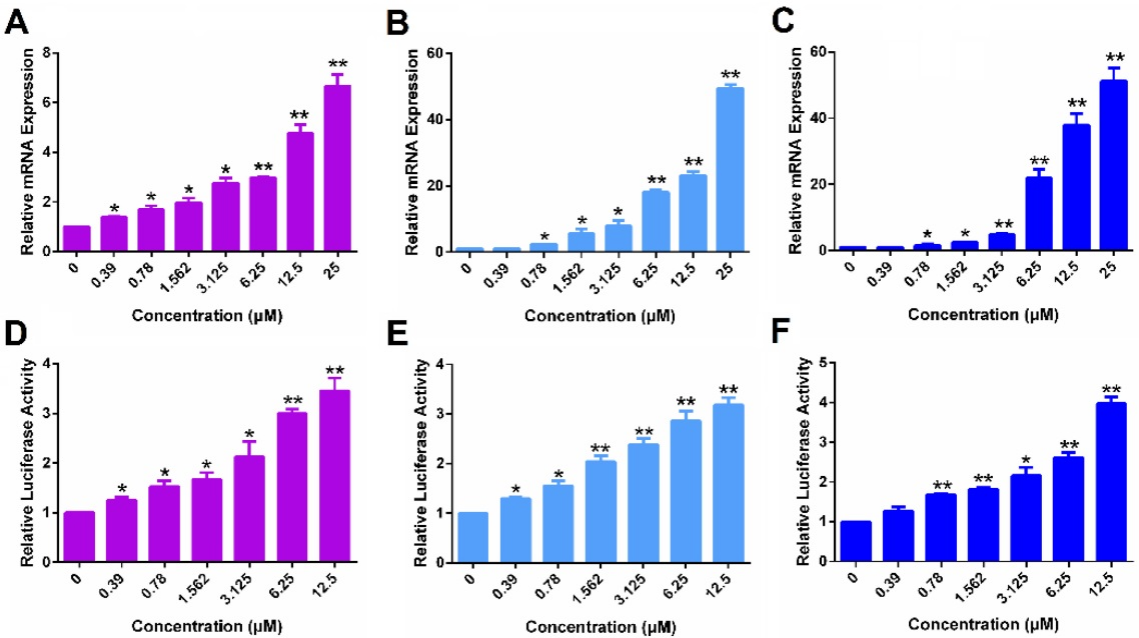
** The function of ERα re-activated by POEG-*co*-PVDSAHA micelles in TNBC cells.** The expression of PGR mRNA induced by POEG-*co*-PVDSAHA micelles in MDA-MB-231 cells (A), HS578T cells (B) and 4T1.2 cells (C). Effect of POEG-*co*-PVDSAHA micelles on ERE expression in MDA-MB-231 cells (D), HS578T cells (E) and 4T1.2 cells (F). The concentrations of SAHA in the micelles are 0, 0.39, 0.78, 1.562, 3.125, 6.25 and 12.5 µM. Data represents the means ± SEM (n=3). * *p*< 0.05, ** *p*< 0.01 versus the control group.

Figure 6 shows that POEG-*co*-PVDSAHA similarly induced the expression of PGR and *ERE*(3)-luciferase reporter in all 3 TNBC cell lines. The above data clearly suggest that POEG-*co*-PVDSAHA well retained the activity of SAHA to induce the expression of functional ERα. We then went on to further examine the ability of POEG-*co*-PVDSAHA as a nanocarrier for codelivery of TAM and the potential synergistic antitumor activity as detailed below.

### Physicochemical characterization of blank and TAM-loaded micelles

**Figure 7:**
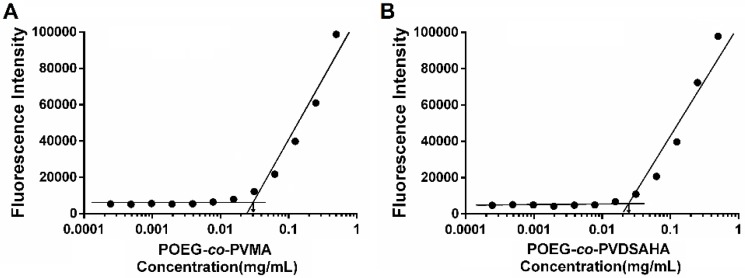
Measurement of CMC of POEG-*co*-PVMA (A) and POEG-*co*-PVDSAHA (B) micelles.

POEG-*co*-PVDSAHA and POEG-*co*-PVMA prodrug micelles were prepared by a film hydration method. The CMC of POEG-*co*-PVDSAHA and POEG-*co*-PVMA micelles were evaluated by fluorescence assay with nile red as a fluorescence probe. As shown in Figure 7, POEG-*co*-PVDSAHA micelles showed slightly decreased CMC values (25 μg/mL) compared with POEG-*co*-PVMA micelles (31 μg/mL). The relatively low CMC could render the micelles stable in the blood circulation.

**Figure 8:**
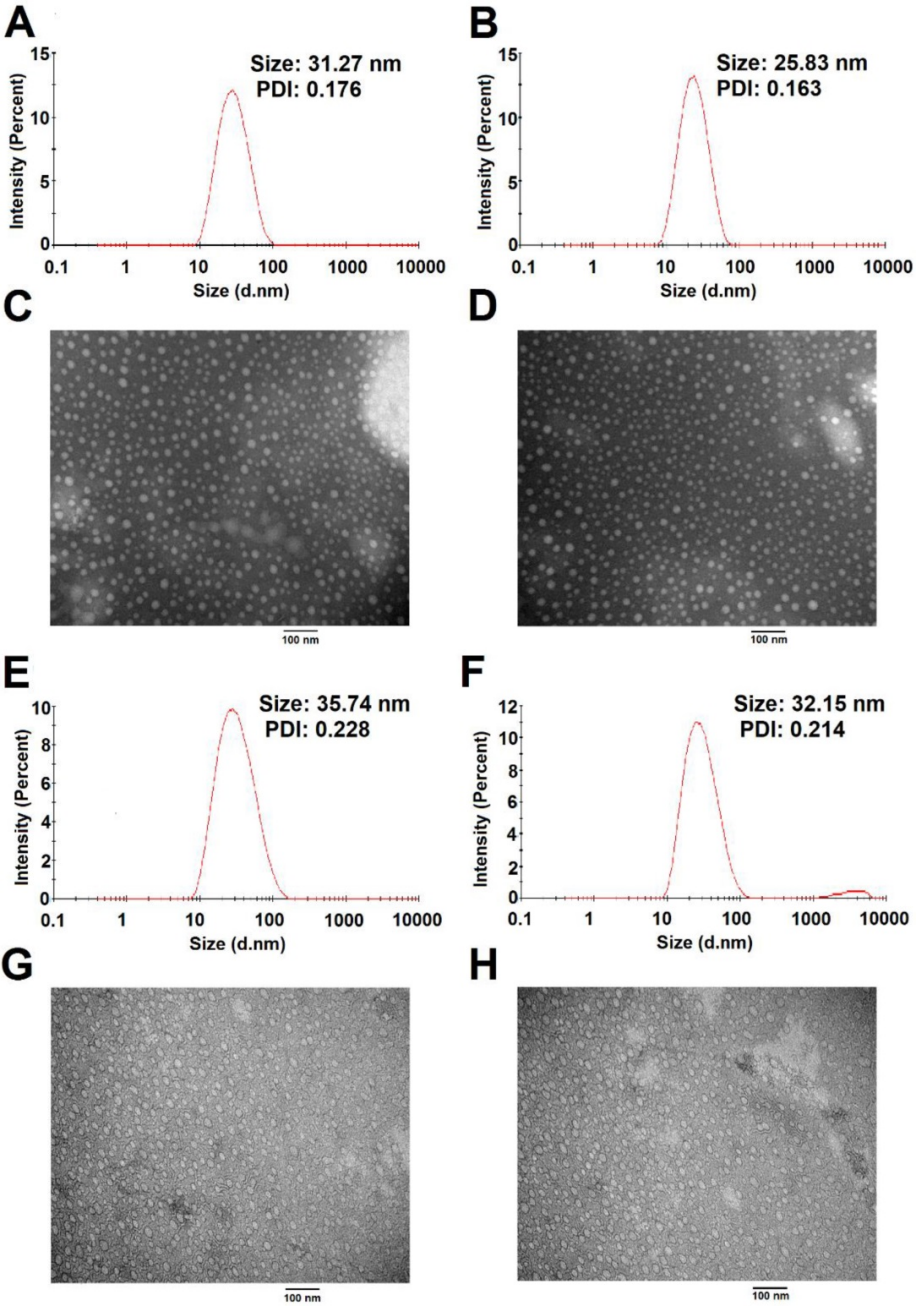
** Size distribution and morphology of POEG-*co*-PVDSAHA (A&C), TAM-loaded POEG-*co*-PVDSAHA (B&D), POEG-*co*-PVMA (E&G) and TAM-loaded POEG-*co*-PVMA (F&H) micelles were measured by DLS and TEM, respectively.** TAM concentration in micelles was kept at 1mg/mL. The mass ratio of carrier/drug was 10/1. Scale bar was 100 nm.

The size distribution and morphology of blank and TAM-loaded micelles were investigated by dynamic light scattering (DLS) and TEM as shown in Figure 8. POEG-*co*-PVDSAHA and POEG-*co*-PVMA micelles could readily form small-sized micelles in aqueous solutions. The average particle sizes of POEG-*co*-PVDSAHA (Figure 8A & C) and POEG-*co*-PVMA (Figure 8E & G) micelles were 31.27 nm and 35.74 nm, respectively. Loading of TAM into both micelles resulted in slight decreases in the sizes of the particles. The average particle sizes of TAM/POEG-*co*-PVDSAHA (Figure 8B & D) and TAM/POEG-*co*-PVMA (Figure 8F & H) micelles were 25.83 nm and 32.15 nm, respectively.

The TAM loading capacity (DLC) and efficiency (DLE) of the TAM/POEG-*co*-PVDSAHA micelles were determined by HPLC. TAM/POEG-*co*-PVDSAHA micelles showed a DLC of 7.3% and a DLE of 80.4%. The stability of the TAM/POEG-*co*-PVDSAHA micelles was evaluated by examining the size changes after exposure to saline, DMEM (2% FBS) and BSA (30 mg/mL) at indicated time intervals. As shown in Figure S5, there were minimal changes in the sizes of TAM/POEG-*co*-PVDSAHA micelles under all conditions within 48 h.

**Figure 9:**
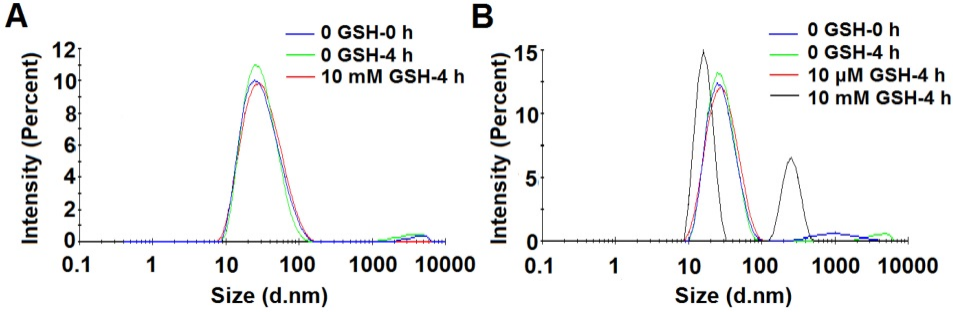
** Size change profiles of POEG-*co*-PVMA (A) and POEG-*co*-PVDSAHA (B) micelles in response to GSH.** The concentration of micelles was 1 mg/mL.

The redox-sensitivity of POEG-*co*-PVDSAHA micelles was evaluated by testing the size changes in response to GSH with POEG-*co*-PVMA micelles as a control (Figure 9). Two concentrations of GSH (10 µM and 10 mM) were used to simulate its concentrations in the blood and inside tumor cells, respectively^2^. POEG-*co*-PVMA micelles were stable in the presence of 10 mM GSH (Figure 9A). In contrast, the size of POEG-*co*-PVDSAHA micelles changed significantly from 25.83 to 258.5 nm after exposure to 10 mM GSH (Figure 9B). No obvious size change of POEG-*co*-PVDSAHA micelles was observed in the presence of 10 μM GSH (Figure 9B).

**Figure 10:**
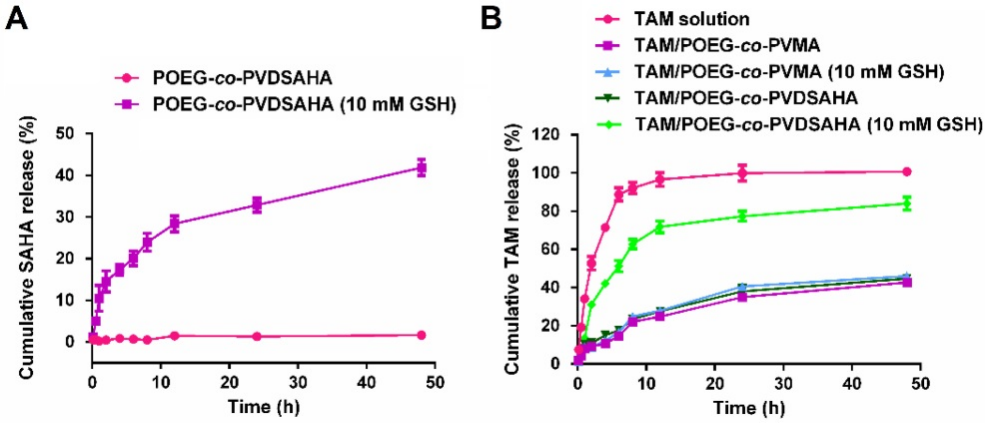
***In vitro* release of SAHA and TAM from POEG-*co*-PVMA and POEG-*co*-PVDSAHA micelles.** (A) Cumulative SAHA release profiles from POEG-*co*-PVDSAHA micelles. (B) TAM release profiles of TAM-loaded POEG-*co*-PVMA and TAM-loaded POEG-*co*-PVDSAHA micelles with free TAM as the control. PBS solution containing 0.5% (w/v) Tween 80 and different concentrations of GSH (0, 10 mM) was used as the release medium. TAM concentration was kept at 1mg/mL. The mass ratio of carrier/drug was 10/1. Data are presented as means ± SEM (n= 3).

*In vitro* release of SAHA and TAM was investigated in PBS solution with or without 10 mM GSH. As shown in Figure 10A, little release of SAHA was observed from the POEG-*co*-PVDSAHA micelles within 48 h in PBS solution without GSH. In contrast, the release of SAHA from POEG-*co*-PVDSAHA micelles with 10 mM GSH was significantly accelerated with more than 40% of SAHA being released at 48 h.

Figure 10B shows the kinetics of TAM release from TAM/POEG-*co*-PVDSAHA and TAM/POEG-*co*-PVMA micelles. Free TAM rapidly diffused across the dialysis bag with greater than 90% of TAM being found outside of dialysis bag within 12 h. TAM-loaded POEG-*co*-PVDSAHA and POEG-*co*-PVMA micelles without GSH showed a slow kinetics of TAM release: only less than 30% TAM was released at 12 h and more than 50% TAM still remained in the micelles within 48 h. Incubation with 10 mM GSH showed minimal impact on the release of TAM from TAM/POEG-*co*-PVMA micelles. In contrast, the release of TAM from TAM/POEG-*co*-PVDSAHA micelles with 10 mM GSH was significantly accelerated with more than 70% of TAM being released at 12 h, which was due to the disassembly of TAM/POEG-*co*-PVDSAHA micelles following the cleavage and release of SAHA from POEG-*co*-PVDSAHA by GSH.

### *In vitro* cytotoxicity

**Figure 11:**
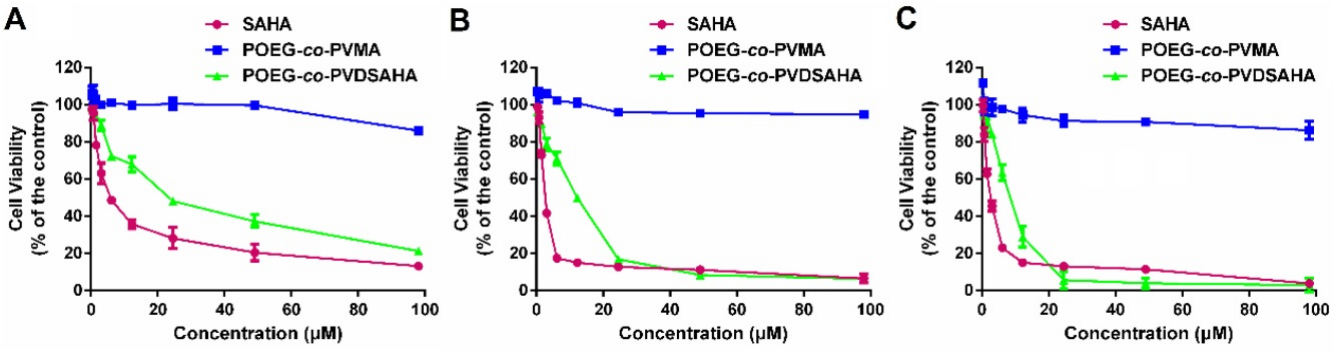
***In vitro* cytotoxicity of POEG-*co*-PVMA and POEG-*co*-PVDSAHA micelles in MDA-MB-231 cells (A), HS578T cells (B) and 4T1.2 cells (C) with free SAHA as the control.** The concentrations of SAHA are 0, 0.742, 1.484, 2.969, 5.938, 11.875, 23.75, 47.5 and 98 μM. The values represent the average of three independent experiments. Data represents the means ± SEM (n=3).

The cytotoxicity of POEG-*co*-PVDSAHA and POEG-*co*-PVMA micelles was evaluated by MTT assay in TNBC cells with free SAHA as control. As shown in Figure 11, POEG-*co*-PVMA micelles were not effective in inhibiting the proliferation of TNBC cells. Meanwhile, free SAHA and POEG-*co*-PVDSAHA micelles inhibited the proliferation of TNBC cells in a SAHA dose dependent manner. POEG-*co*-PVDSAHA micelles showed lower cytotoxicity comparing with free SAHA at the equivalent concentrations of SAHA, which might be due to only partial release of SAHA from the POEG-*co*-PVDSAHA polymer within a short period of treatment.

**Figure 12:**
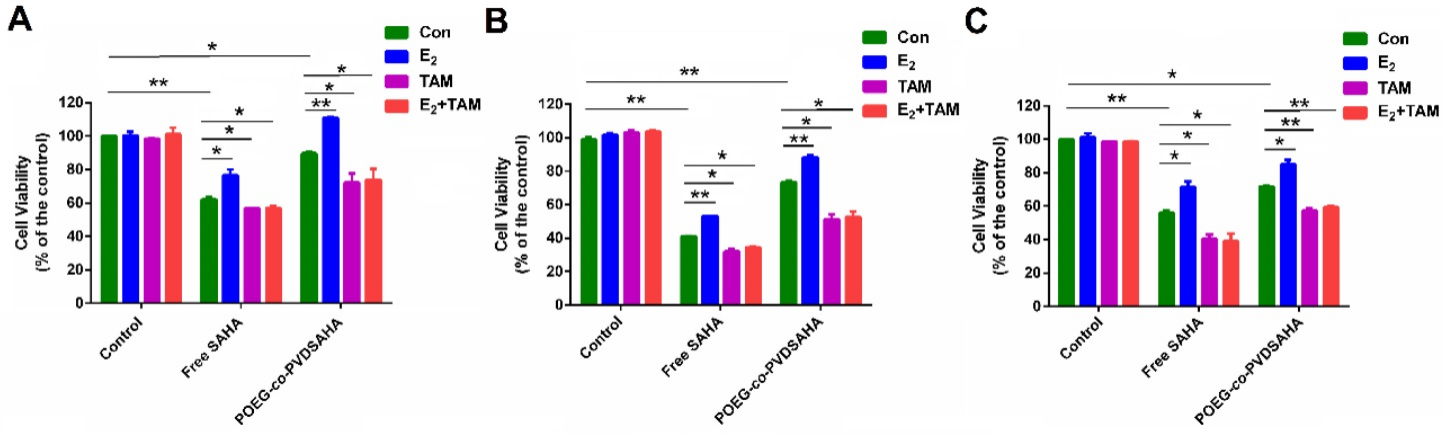
** Cellular viability in response to E_2_ and TAM in MDA-MB-231 cells (A), HS578T cells (B) and 4T1.2 cells (C).** In this study, the concentration of E_2_ and TAM was 10 nM and 3 µM, respectively. The concentration of SAHA was equivalent to that of SAHA in POEG-*co*-PVDSAHA micelles (carrier: TAM, 10:1, m/m). The values represent the average of three independent experiments. Data represents the means ± SEM (n=3).

Figure 12A shows the impact of POEG-*co*-PVDSAHA or free SAHA co-treatment on the response of MDA-MB-231 cells to E2 and TAM. Control cells did not show any response to either E2 (10 nM) or TAM (3 µM) at the concentration used. Treatment with SAHA or POEG-*co*-PVDSAHA (6 µM) led to a significant inhibition of the proliferation of MDA-MB-231 cells. However, co-treatment with E2 led to a partial attenuation of the proliferation inhibition by SAHA. Nonetheless, the stimulating effect of E2 was completely abolished by TAM and the level of cytotoxicity of combination of TAM with SAHA or POEG-*co*-PVDSAHA in the presence of E2 was even higher than that of SAHA alone in the absence of E2. Similar results were shown in HS578 (Figure 12B) and 4T1.2 (Figure 12C) cell lines.

**Figure 13:**
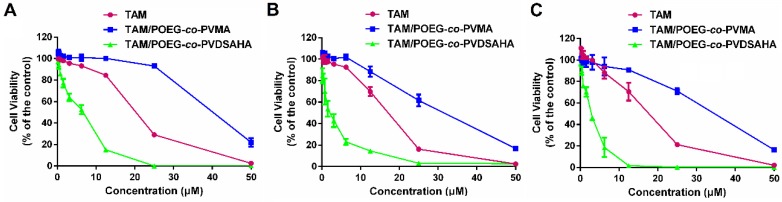
***In vitro* cytotoxicity of TAM/POEG-*co*-PVMA and TAM/POEG-*co*-PVDSAHA micelles in MDA-MB-231 cells (A), HS578T cells (B) and 4T1.2 cells (C) with free TAM as the control.** The concentrations of TAM are 0, 0.39, 0.78, 1.562, 3.125, 6.25, 12.5, 25 and 50 μM. The mass ratio of carrier/drug was 10/1. The values represent the average of three independent experiments. Data represents the means ± SEM (n=3).

Figure 13 shows the cytotoxicity of various concentrations of free TAM, TAM/POEG-*co*-PVMA and TAM/POEG-*co*-PVDSAHA micelles in TNBC cells. TAM/POEG-*co*-PVMA micelles showed less inhibitory effect on the proliferation of TNBC cells compared to free TAM and TAM/POEG-*co*-PVDSAHA micelles. TAM/POEG-*co*-PVDSAHA micelles were more effective than free TAM at equivalent concentrations of TAM in TNBC cells. Table S1 shows the IC_50_ values of free TAM, carrier alone and TAM-loaded micelles. The IC_50_ value of TAM/POEG-*co*-PVDSAHA micelles (2.94 µM for TAM, 5.76 µM for SAHA) was much lower than that of free TAM (17.05 µM) and POEG-*co*-PVDSAHA micelles (7.74 µM for SAHA) in 4T1.2 cells. This was also the case in the HS578T and MDA-MB-231 cells, indicating a synergistic antitumor effect of POEG-*co*-PVDSAHA micelles and co-delivered TAM.

### Near-infrared fluorescence optical imaging

**Figure 14:**
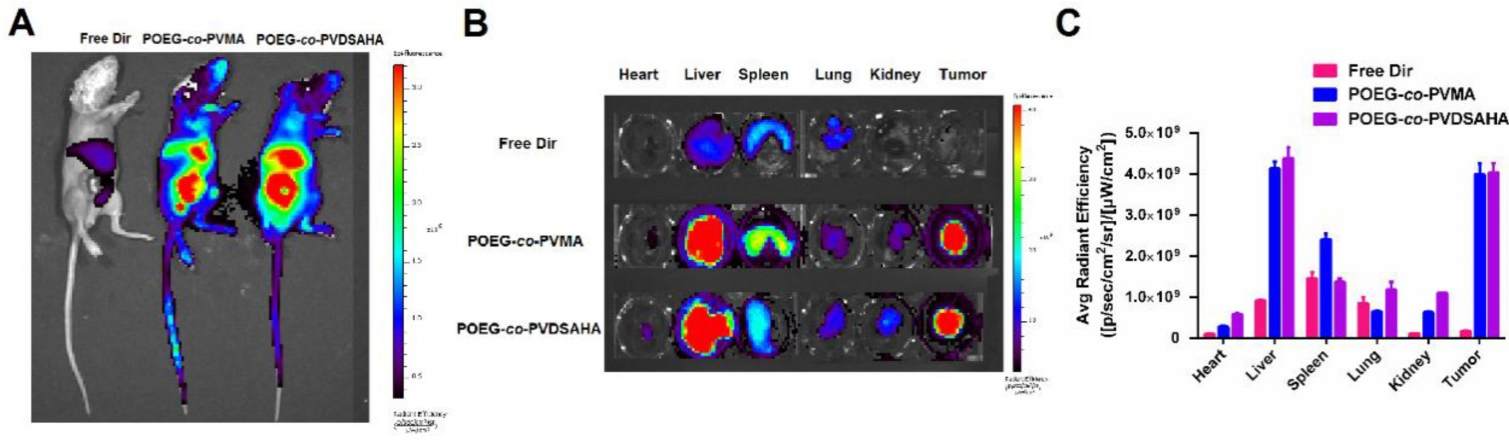
** Biodistribution of POEG-*co*-PVMA and POEG-*co*-PVDSAHA micelles in 4T1.2 xenograft-bearing mice.** (A) *In vivo* and (B) *ex vivo* NIRF imaging of DiR-loaded POEG-*co*-PVMA and POEG-*co*-PVDSAHA micelles with free Dir as the control after 24 h. (C) Quantitative fluorescence intensities of tumors and major organs from *ex vivo* images. The DiR concentration was 0.4 mg/mL and the mass ratio of carrier/Dir was 10/1. The injection volume was 200 µL. The values represent the average of three independent experiments. Data represents the means ± SEM (n=3).

*In vivo* biodistribution of POEG-*co*-PVDSAHA and POEG-*co*-PVMA micelles was evaluated by near-infrared fluorescent optical imaging in a 4T1.2 xenograft model. Dir, a highly penetrating fluorescence dye, was loaded into the POEG-*co*-PVDSAHA and POEG-*co*-PVMA micelles for tissue imaging with the free Dir as a control. Figure 14A shows that Dir-loaded POEG-*co*-PVDSAHA and POEG-*co*-PVMA micelles largely concentrated at tumor and site at 24 h. Significant accumulation was also found in the liver. No obvious signal was observed at the tumor site with free Dir treatment.

For* ex vivo* imaging and quantification, major organs and tumors were harvested at 24 h post-injection. Consistent with the results from whole body imaging, large amounts of signals were observed in the tumor tissues treated with Dir loaded into either POEG-*co*-PVDSAHA or POEG-*co*-PVMA micelles. The signals in the tumor tissues were significantly higher than those in heart, lungs and kidneys (Figure 14B & C). The signals in the liver were similar to those in the tumors, which is likely due to the nonspecific uptake of nanoparticles by RES. Free Dir was largely found in liver, spleen, and lungs with little accumulation in the tumors.

### *In vivo* therapeutic efficacy

**Figure 15:**
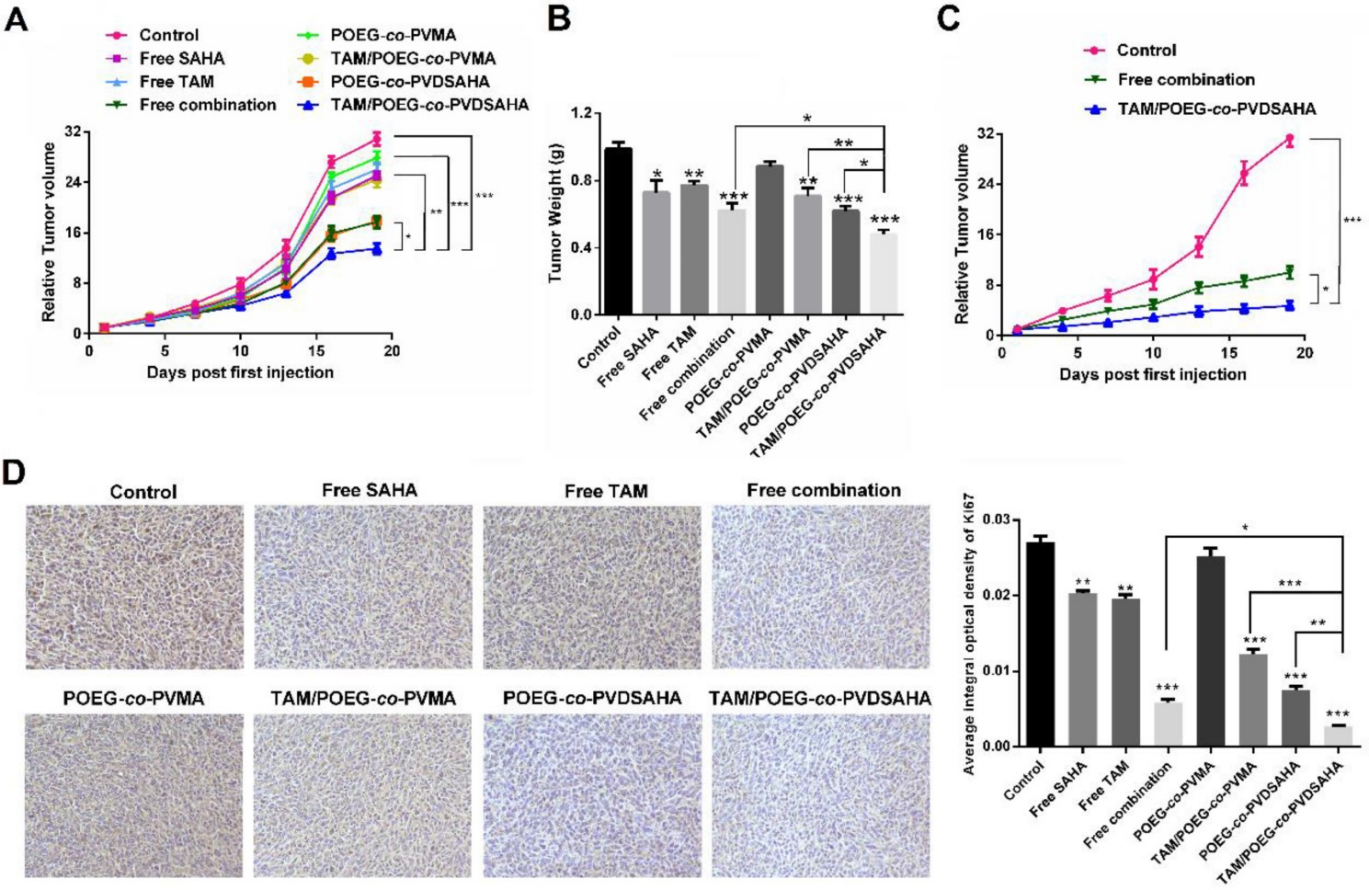
***In vivo* antitumor activity of various formulations in 4T1.2 tumor model.** (A) Tumor sizes were plotted as relative tumor volumes at different time point. TAM dose was 10 mg/kg and the mass ratio of carrier/drug was 10/1. The injection volume was 50 µL. Data represents the means ± SEM (n=5). * *p*< 0.05; ** *p*< 0.01. (B) The tumor weight of each group at day 20. Data represents the means ± SEM (n=5). * *p*< 0.05; ** *p*< 0.01 (all treatment groups versus control group). (C) Tumor sizes were plotted as relative tumor volumes at different time point. TAM dose was 20 mg/kg and the mass ratio of carrier/drug was 10/1. The injection volume was 50 µL. Data represents the means ± SEM (n=5). * *p*< 0.05; ** *p*< 0.01. (D) Ki67 staining of tumor tissues collected on day 20 using immunohistochemical analysis.

Figure S6 shows that treatment of 4T1.2 tumor model with free SAHA and POEG-*co*-PVDSAHA led to increased expression of ERα and PGR at mRNA level, which was consistent with the results *in vitro*. Figure 14A & B show the *in vivo* antitumor activity of free SAHA, free TAM, combination of free SAHA and TAM, POEG-*co*-PVMA, TAM/POEG-*co*-PVMA, POEG-*co*-PVDSAHA and TAM/POEG-*co*-PVDSAHA at a TAM dosage of 10 mg kg^-1^ and a SAHA dose of 13.9 mg kg^-1^, respectively. Free TAM or SAHA showed minimal effect in inhibiting the growth of 4T1.2 tumor. POEG-*co*-PVMA alone also had minimal impact on the tumor growth and delivery of TAM via POEG-*co*-PVMA resulted in no improvement in antitumor activity. The combination of the two free drugs led to a significant improvement in antitumor activity. A comparable level of antitumor activity was also observed for POEG-*co*-PVDSAHA alone. Delivery of TAM via POEG-*co*-PVDSAHA nanocarrier led to the best therapeutic outcome compared to other treatment groups. Doubling the dose of TAM and SAHA led to a further improvement in the antitumor activity (Figure 15C). Nonetheless, TAM/POEG-*co*-PVDSAHA remained significantly more effective than the free drug combination in inhibiting the growth of 4T1.2 tumor (Figure 15C).

We further examined the antitumor activities of different treatments via Ki67 immunohistochemical staining of the tumor tissues that were collected at the completion of the therapy study. As shown in Figure 15D, the Ki67 index of tumor tissues after treatment with free TAM, SAHA, POEG-*co*-PVMA or TAM/POEG-*co*-PVMA was slightly decreased compared to control group. In addition, treatment with combination of free SAHA and TAM led to a significant reduction in tumor cell proliferation, which was comparable to the POEG-*co*-PVDSAHA group. By comparing all the treatments, TAM/ POEG-*co*-PVDSAHA was the most effective in inhibiting the proliferation of tumor cells, which was consistent with the data in Figure 15A.

To investigate the potential toxicity associated with different treatments, serum levels of creatinine, AST and ALT in each group were tested. As shown in Figure S7, there were no significant increases in the serum levels of these biomarkers in any of the treatment groups compared to control group. In addition, the body weights of the mice in each treatment group were similar to those in control group (Figure S8 & S9). The results suggest that all the treatments were well tolerated at the doses used.

## Discussion

TNBCs account for about 15% of all breast cancers in the clinic. For this subset of breast cancer patients, there are no receptor targets (ER, PR and HER2), therefore, the currently available hormone therapy (such as TAM) and HER2-targeted therapy (such as *Herceptin)* are not applicable. TNBCs are much more aggressive, more invasive and have poor prognosis^44^. Some studies have shown that ER gene silence is partially due to the histone deacetylation and HDAC inhibitors can reactivate the expression of functional ER^9^. Among all the HDAC inhibitors, SAHA is the first pan-HDAC inhibitor approved by FDA and has shown promising antitumor efficacy in different types of cancers. In this study, the effect of SAHA on the re-expression of ERα was investigated. Our data showed that SAHA could reactivate the ERα expression in several TNBC cell lines (4T1.2, MDA-MB-231 and HS578T cells) at both mRNA and protein levels (Figure 1). The reactivated ERα was functional as shown by E2-mediated induction of PGR and a luciferase reporter driven by a 3X-ERE-promoter (Figure 2). More importantly, SAHA re-sensitized all the 3 TNBC cell lines to E2 and TAM with respect to stimulation/inhibition of cell proliferation and combination of SAHA and TAM demonstrated synergistic antitumor activity both *in vitro* and *in vivo*.

We have also developed a SAHA prodrug-based nanocarrier to facilitate the *in vivo* codelivery of SAHA and TAM. POEG-*co*-PVDSAHA was designed via incorporation of SAHA to the polymer backbone through a disulfide linkage. In addition to its intrinsic antitumor activity, SAHA serves as part of the hydrophobic domain of the amphiphilic polymer to facilitate the formation of stable micelles as well as the loading of TAM into the micelles. It has been reported that GSH is present at very low concentrations (2-20 μM) in the blood or extracellular matrices but its concentrations (0.5-10 mM) were significantly increased inside various types of tumor cells^2^, ^45^. Our data (Figure 9 & 10) showed that SAHA was stably incorporated into the POEG-*co*-PVDSAHA micelles at 10 µM of GSH but became rapidly cleaved and released from the carrier upon exposure to 10 mM GSH, which subsequently led to rapid release of loaded TAM. Therefore, TAM/POEG-*co*-PVDSAHA micelles are expected to be stable in the blood but become destabilized upon reaching tumor cells to facilitate the release of both SAHA and the loaded TAM. TAM/POEG-*co*-PVDSAHA micelles also have low CMC value, which further enhances its stability upon dilution in the blood stream. This, together with the small sizes of the micelles, is likely to contribute significantly to the effective targeting to the tumors as evident from the imaging study (Figure 14).

POEG-*co*-PVDSAHA polymer well retained the pharmacological activity of SAHA in reactivating the expression of functional ERα in all 3 TNBC cell lines (Figure 5 & 6). The cytotoxicity of TAM/POEG-*co*-PVDSAHA was significantly higher than that of free TAM and TAM formulated in the control carrier in the 3 TNBC cell lines (Figure 13), suggesting a synergy between TAM and the SAHA that is released from POEG-*co*-PVDSAHA. Incorporation of TAM into POEG-*co*-PVDSAHA micelles also led to a significant improvement in the overall antitumor activity *in vivo*, much more effectively than other treatments, particularly the combination of free SAHA and TAM (Figure 15). The enhanced antitumor activity of TAM/POEG-*co*-PVDSAHA is likely attributed to the improved codelivery of SAHA and TAM as shown in a preliminary biodistribution study (Figure S10). Particularly POEG-*co*-PVDSAHA shall help to improve the delivery of the prodrug and provide sustained release of pharmacologically active SAHA as evident from the significantly enhanced antitumor activity of POEG-*co*-PVDSAHA compared to free SAHA. The synergy between TAM and the released SAHA from POEG-*co*-PVDSAHA may have also contributed to the overall antitumor activity. It should be noted that free TAM was essentially not active *in vivo*. An improvement in its delivery via a control nanocarrier led to no improvement in antitumor activity. This is consistent with the notion that endocrine therapy alone such as TAM is not effective for TNBCs^46^. Therefore, the synergy noticed in TAM/POEG-*co*-PVDSAHA is likely mediated by the reactivated ERα following treatment with POEG-*co*-PVDSAHA. More studies are needed to better understand the underlying mechanism for the combination therapy.

In summary, we have shown that POEG-*co*-PVDSAHA nanoparticles was effective in inducing reactivation of functional ERα in both human and murine TNBC cell lines, leading to resensitization to TAM. POEG-*co*-PVDSAHA was efficient to encapsulate TAM. Moreover, co-delivery of TAM using our POEG-*co*-PVDSAHA nanoparticles showed synergy in inhibiting the proliferation of TNBC cells both *in vitro* and *in vivo*. Our prodrug-based co-delivery system may provide a simple but highly effective strategy to re-sensitize TNBCs to chemotherapy.

## Supplementary Material

Supplementary figures and tables.

## Abbreviations

TNBCs: triple negative breast cancers
TAM: tamoxifen
SAHA: vorinostat
POEG-*co*-PVDSAHA: vorinostat-containing polymeric nanocarrier
RAFT: reversible addition-fragmentation transfer
ER: estrogen receptor
POEG-*co*-PVMA: TAM loaded into a pharmacologically inert control carrier
HDACs: histone deacetylases
HATs: histone acetyltransferases
FDA: Food and Drug Administration
PR: progesterone receptor
HER2: human epidermal growth factor 2
DMEM: dulbecco's modified eagle medium
FBS: fetal bovine serum
MTT: 3-(4,5-dimethylthiazol-2-yl)-2,5-diphenyl tetrazolium bromide
DMSO: dimethyl sulfoxide
DPBS: dulbecco's phosphate buffered saline
PVDF: polyvinylidene fluoride
AST: aspartate aminotransferase
ALT: alanine transaminase
TEM: transmission electron microscopy
CMC: critical micelle concentration
DCM: dichloromethane
GSH: glutathione
TBST: tris-buffer saline containing 0.05% Tween-20
ERE: estrogen response element
ESI: electrospray ionization
SEM: means ± standard error of means
PGR: progesterone receptor gene
CI: combination index
DLS: dynamic light scattering
DLC: loading capacity
DLE: loading efficiency
