# Supplementary material for: Sensitizing Triple Negative Breast Cancer to Tamoxifen Chemotherapy via a Redox-Responsive Vorinostat-containing Polymeric Prodrug Nanocarrier
Source: Theranostics. 2020 Jan 20;10(6):2463–78. doi: 10.7150/thno.38973 (PMC7052901; doi:10.7150/thno.38973)
Supplement: Supplementary file 1 — Supplementary figures and tables. [file thnov10p2463s1.pdf]

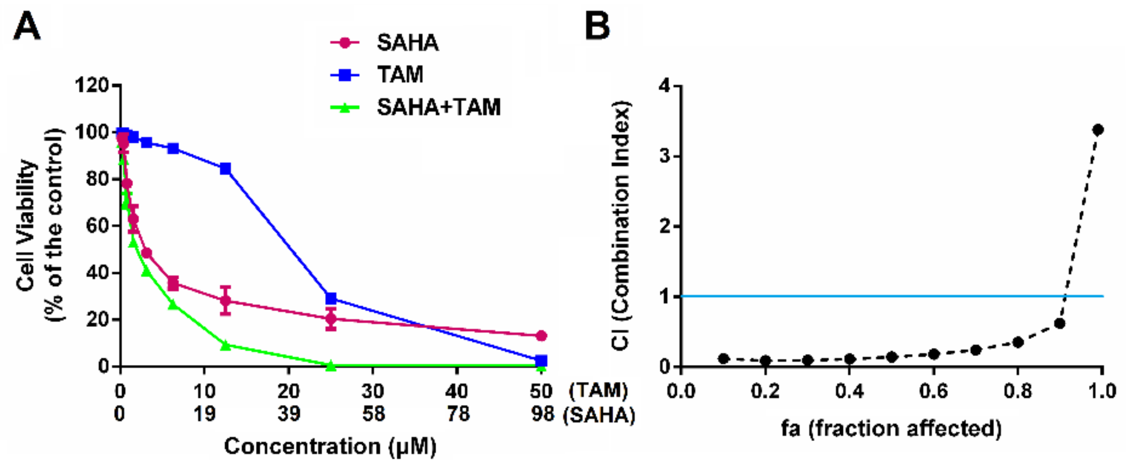

Figure S1. Synergistic antitumor effect between SAHA and TAM on MDA-MB-231 cell proliferation. (A) Dose-response study of a fixed ratio combination of SAHA (0, 0.742, 1.484, 2.969, 5.938, 11.875, 23.75, 47.5 and 98 μM) and TAM (0, 0.39, 0.78, 1.562, 3.125, 6.25, 12.5, 25 and 50 μM) against MDA-MB-231 cells. (B) fa-CI plot in which fa and CI indicate fraction affected and combination index, respectively. CI<1, CI=1, and CI>1 denote synergistic, additive, and antagonistic interaction, respectively. Each data represents the means ± SEM of triplicate experiments.

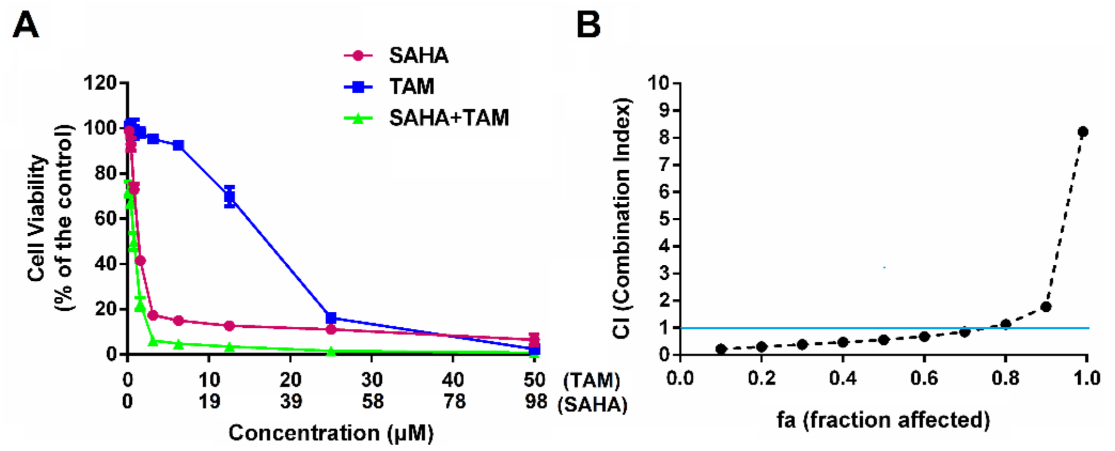

Figure S2. Synergistic antitumor effect between SAHA and TAM on HS578T cell proliferation. (A) Dose-response study of a fixed ratio combination of SAHA (0, 0.742, 1.484, 2.969, 5.938, 11.875, 23.75, 47.5 and 98 μM) and TAM (0, 0.39, 0.78, 1.562, 3.125, 6.25, 12.5, 25 and 50 μM) against HS578T cells. (B) fa-CI plot in which fa and CI indicate fraction affected and combination index, respectively. CI<1, CI=1, and CI>1 denote synergistic, additive, and antagonistic interaction, respectively. Each data represents the means ± SEM of triplicate experiments.

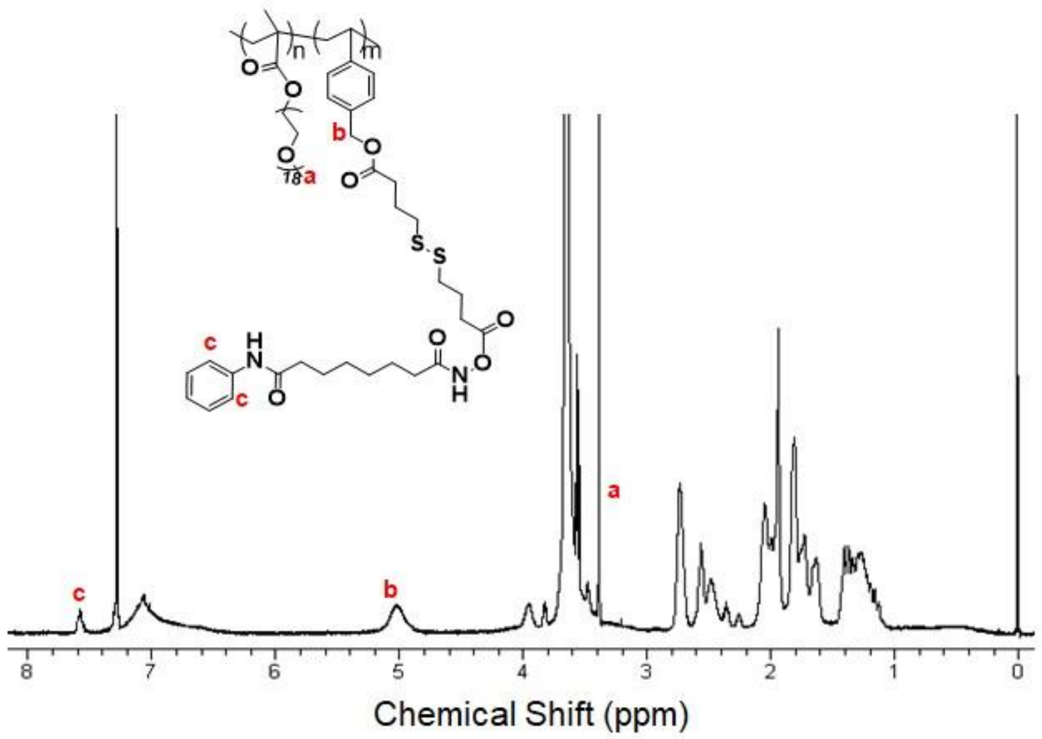

Figure S3. [1]**H NMR spectrum of the POEG-*co*-PVDSAHA polymer in CDCl$_3$.**

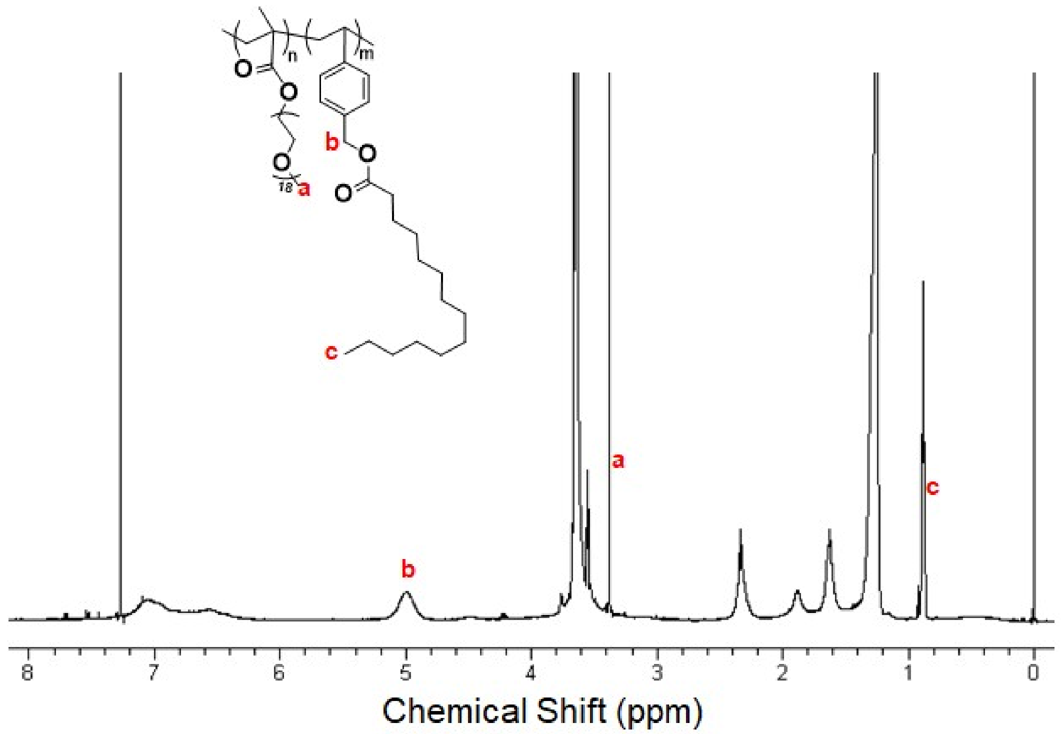

Figure S4. [1]**H NMR spectrum of the POEG-*co*-PVMA polymer in CDCl$_3$.**

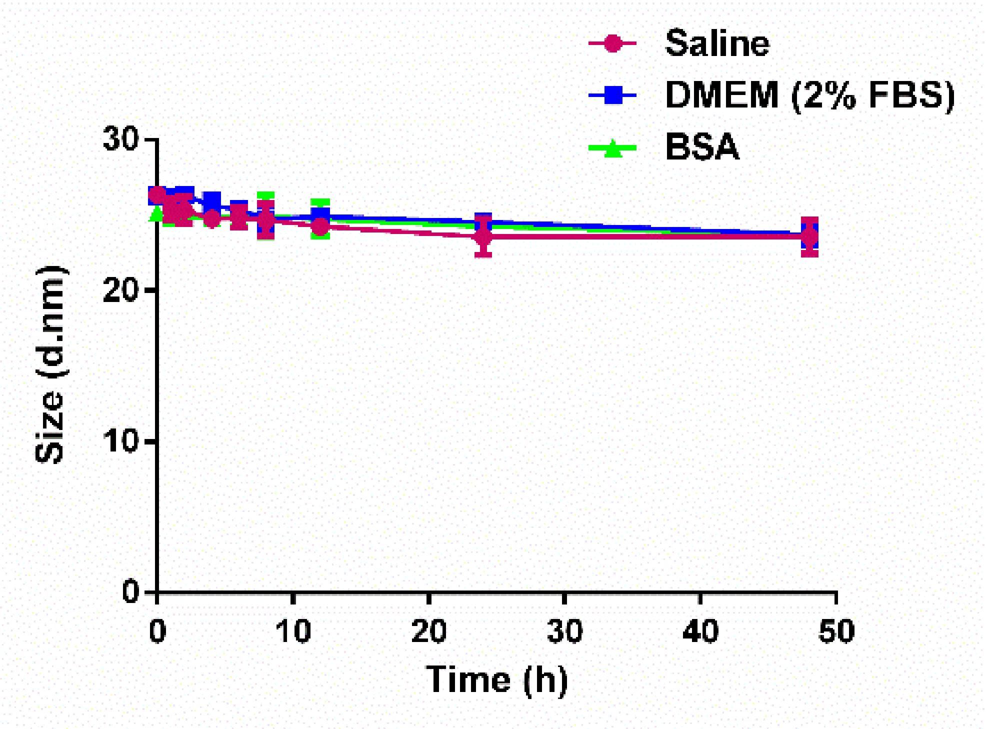

Figure S5. The stability of TAM-loaded POEG-*co*-PVDSAHA micelles in saline, DMEM (2% FBS) and BSA (30 mg/mL). TAM concentration in micelles was kept at 1mg/mL. The mass ratio of carrier/drug was 10/1.

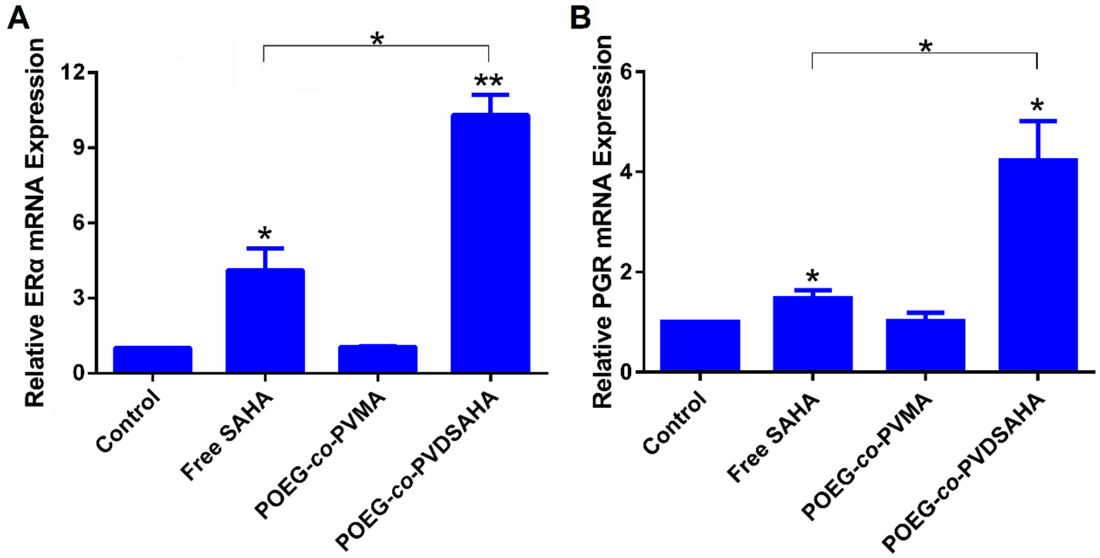

Figure S6. The expression of ERα (A) and PGR (B) induced by Free SAHA, POEG-*co*-PVMA and POEG-*co*-PVDSAHA micelles in 4T1.2 tumor model. SAHA dose was 13.9 mg/kg. The injection volume is 50 μL. Data represents the means ± SEM (n=5). * $p < 0.05$, ** $p < 0.01$.

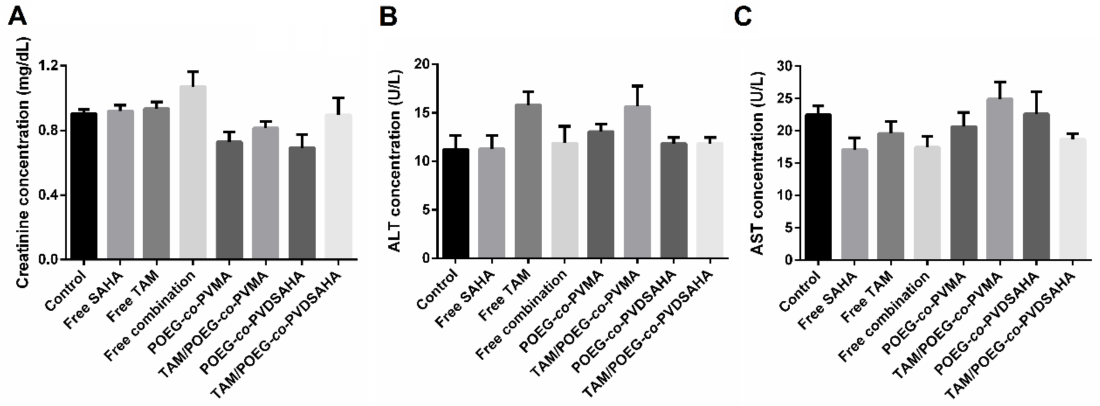

Figure S7. The content of creatinine (A), ALT (B) and AST (C) in the serum of each group *in vivo*. Each data represents the means ± SEM of triplicate experiments. TAM dose was 10 mg/kg and SAHA dose was 13.9 mg/kg. The mass ratio of carrier/drug was 10/1. The injection volume is 50 μL.

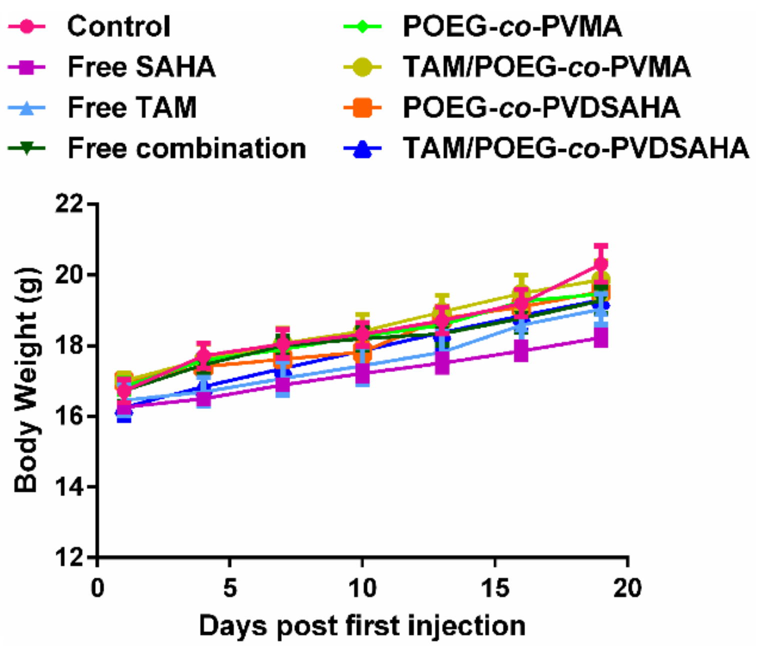

Figure S8. Changes of body weight in 4T1.2 tumor model with different treatments. TAM dose was 10 mg/kg and SAHA dose was 13.9 mg/kg. The mass ratio of carrier/drug was 10/1. The injection volume is 50 μL.

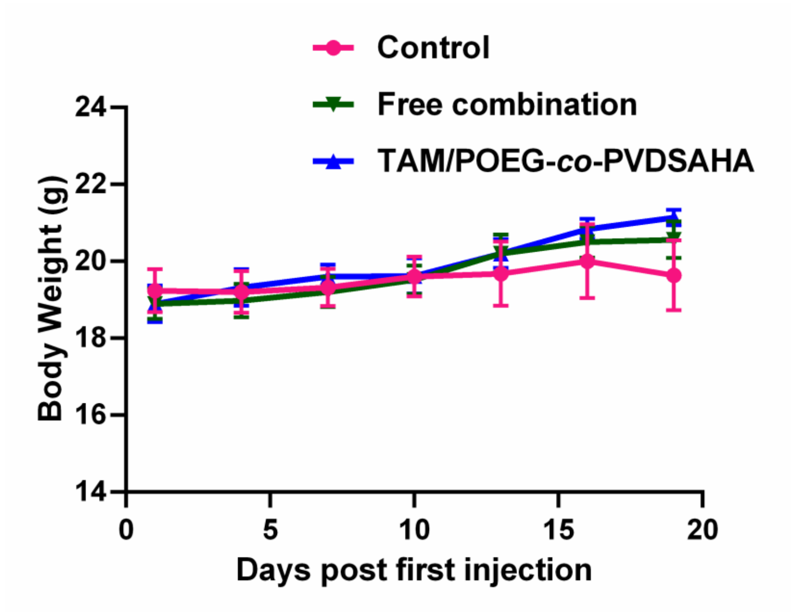

Figure S9. Changes of body weight in 4T1.2 tumor model with different treatments. TAM dose was 20 mg/kg and SAHA dose was 27.8 mg/kg. The injection volume is 50 μL.

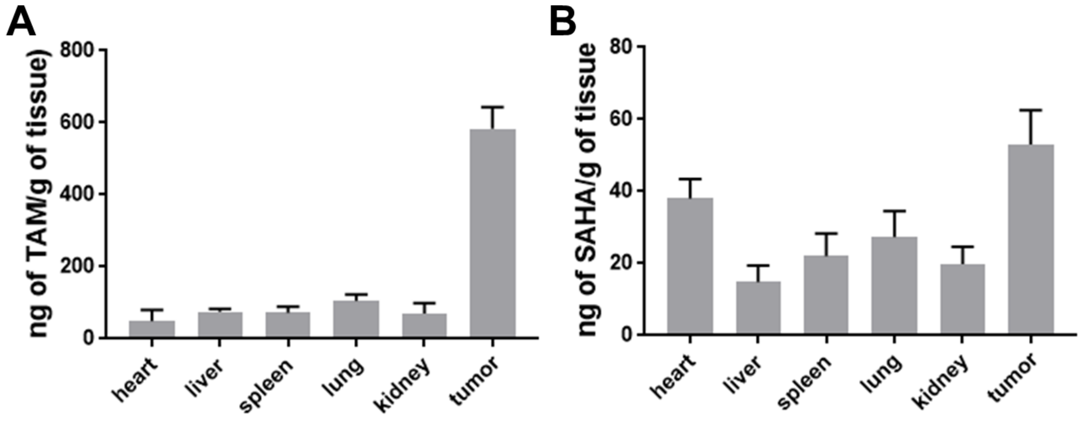

Figure S10. Tissue distribution of TAM (A) and SAHA (B) in 4T1.2 tumor-bearing BALB/c mice at 24 h following i.v. administration of TAM-loaded POEG-*co*-PVDSAHA micelles.

Table S1. IC_{50} values for SAHA, POEG-*co*-PVMA and POEG-*co*-PVDSAHA prodrug micelles, TAM, TAM-loaded POEG-*co*-PVMA and POEG-*co*-PVDSAHA micelles in TNBC cell lines.

Treated groups	IC_{50} (μM)		
	4T1.2 cells	HS578T cells	MDA-MB-231 cells
SAHA	1.66	2.25	3.46
POEG-*co*-PVMA	>50	>50	>50
POEG-*co*-PVDSAHA	7.74 (SAHA)	11.62 (SAHA)	28.93 (SAHA)
TAM	17.05	15.92	20.08
TAM/POEG-*co*-PVMA	42.32 (TAM)	39.71 (TAM)	45.63 (TAM)
TAM/POEG-*co*-PVDSAHA	2.94 (TAM)<br>5.76 (SAHA)	2.57 (TAM)<br>5.03 (SAHA)	6.46 (TAM)<br>12.66 (SAHA)
